# Yellow Mealworm Larvae (*Tenebrio molitor*) Fed Mycotoxin-Contaminated Wheat—A Possible Safe, Sustainable Protein Source for Animal Feed?

**DOI:** 10.3390/toxins11050282

**Published:** 2019-05-21

**Authors:** Carlos Ochoa Sanabria, Natacha Hogan, Kayla Madder, Cedric Gillott, Barry Blakley, Martin Reaney, Aaron Beattie, Fiona Buchanan

**Affiliations:** 1 Department of Animal and Poultry Science, University of Saskatchewan, 51 Campus Drive, Saskatoon, SK S7N 5A8, Canada; carlos.ochoa7@usask.ca (C.O.S.); natacha.hogan@usask.ca (N.H.); kayla.madder@usask.ca (K.M.); 2 Toxicology Centre, University of Saskatchewan, 44 Campus Drive, Saskatoon, SK S7N 5B3, Canada; 3 Department of Biology, University of Saskatchewan, 112 Science Place, Saskatoon, SK S7N 5E2, Canada; cedric.gillott@usask.ca; 4 Western College of Veterinary Medicine, University of Saskatchewan, Saskatoon, SK S7N 5B4, Canada; brb237@mail.usask.ca; 5 Department of Plant Sciences, University of Saskatchewan, 51 Campus Drive, Saskatoon, SK S7N 5A8, Canada; mjr997@mail.usask.ca (M.R.); aaron.beattie@usask.ca (A.B.)

**Keywords:** deoxynivalenol, yellow mealworm, *Fusarium*

## Abstract

The aim of this study was to determine the potential for accumulation of deoxynivalenol (DON) in yellow mealworm larvae (*Tenebrio molitor*) reared on high DON *Fusarium*-infected wheat and investigate the effects on production, survival and nutritional traits. Wheat containing 200 μg/kg DON was used as the control diet. A different source of wheat was sorted into six fractions and mixed to obtain low (2000 μg/kg), medium (10,000 μg/kg) and high (12,000 μg/kg) levels of DON. Each diet was replicated five times with 300 or 200 mealworms per replicate for the feeding and breeding trials, respectively. Trial termination occurred when the first two pupae were observed (32–34 days). There was no difference in the concentrations of DON detected in the larvae between diets that ranged from 122 ± 19.3 to 136 ± 40.5 μg/kg (*p* = 0.88). Excretion of DON was 131, 324, 230 and 742 μg/kg for control, low, medium and high, respectively. Nutritional analysis of larvae showed maximum crude protein of 52% and crude fat of 36%. Ash, fiber, chitin, fatty-acids and amino-acid content were consistent across diets. Survival was greater than 96% for all life stages and average daily gain ranged from 1.9 ± 0.1 to 2.1 ± 0.1 mg/day per mealworm. Larvae accumulated low levels of DON from *Fusarium*-infected wheat diets suggesting contaminated wheat could be used to produce a sustainable, safe protein source.

## 1. Introduction

*Fusarium* mycotoxins in food and feed are a recurring problem for crop and animal production worldwide. Several *Fusarium* species are widespread fungal pathogens affecting cereals and stored grains and are causative agents of Fusarium Head Blight (FHB) disease in wheat [1]. *Fusarium* produces secondary metabolites, also known as mycotoxins, which can downgrade the nutritive, physical, and chemical qualities of grain due to the presence of *Fusarium*-damaged kernels (FDK) [2]. In 2014, it was estimated that two million metric tonnes of wheat and durum were downgraded to salvage (greater than 10% FDK) costing 600 million dollars to Canada’s economy. Two years later, approximately one billion dollars in losses were associated with widespread *Fusarium* infection in Western Canadian wheat crops [3].

*Fusarium graminearum* and *F. culmorum* are the two most prevalent *Fusarium* species in the Canadian prairie provinces and are considered the most harmful due to their ability to produce multiple mycotoxins, primarily deoxynivalenol (DON) [1]. In years with high *Fusarium* infection, farmers are challenged to sell their grain if the level of DON in animal feed would exceed the regulatory limit 1000–5000 μg/kg established by the Canadian Food Inspection Agency (CFIA) [4]. Consequently, grain with significant amounts of FDK is either blended with uncontaminated or low FDK grain, or condemned to salvage grain with no commercial value.

Several strategies have been developed to mitigate the negative impact of mycotoxins in the feed industry. For example, decreasing the amount of FDK via cleaning or neutralizing mycotoxins to improve the grain quality can reduce the amount of salvage grain and improve the commercial value. Near-infrared transmittance (NIT) technology, for instance, is capable of separating out individual FDK to improve the quality and value of *Fusarium* downgraded grain [5]. Alternatively, mycotoxin binders can be incorporated into feed to adsorb and deactivate certain mycotoxins, preventing uptake and toxicity in animals [6]. However, the lack of highly effective disease controls measures, such as genetic resistance or fungicides, and the large impact environmental conditions have on the development of FHB, makes this disease extremely difficult to control. Industry is therefore challenged to find novel and more efficient ways to inactivate/detoxify these harmful compounds as well as to reduce economic losses to the grain sector.

Historically, insects have been an important part of the human diet. Enthomophagy, the consumption of edible insects by humans, was an ancient practice from the palaeolithic era, but is still practiced by many ethnic comunities worldwide [7]. Edible insects contain high nutritional value and are critical to supply the world’s growing population, even when reared on organic wastes [8]. For example, yellow mealworm larvae (*Tenebrio molitor*) have the capacity to recycle low value organic materials and convert them into a source of protein. The nutritional value of larvae fed waste material was reported as 48% crude protein, 38% crude fat, 5% crude fiber and 3% ash [8,9].

A study by Guo et al. [10] demonstrated that yellow mealworm larvae preferred wheat kernels artificially infected with *Fusarium proliferatum, F. poae* and *F. culmorum* over uninfected kernels. These larvae had increased body weight with undetectable levels of DON and zearalenone (ZEN); however, there was high mortality in larvae fed *F. culmorum* [10]. Van Broekhoven et al. [11] reported no effect on survival when yellow mealworm larvae consumed concentrations of DON from either a DON-spiked (8000 µg/kg), or a natural *Fusarium*-infected wheat sources (4900 µg/kg DON); however, DON was excreted in frass at a proportion of 41% and 14%, respectively. These findings suggest that yellow mealworm larvae may detoxify or biotransform DON into derivative forms or unknown metabolites. Based on these initial reports, there is potential for yellow mealworm larvae to be cultivated on mycotoxin-contaminated wheat to produce a safe, alternative source of protein for animal feed. The goals of this study were to determine the potential accumulation of DON in yellow mealworm larvae reared on *Fusarium*-infected wheat containing high levels of DON, and investigate the effects of DON exposure on production, survival and nutritional traits of larvae.

## 2. Results

### 2.1. Fusarium Species Identification and Mycotoxin Levels

*Fusarium graminearum* was identified as the species infecting the wheat source used to formulate the low, medium and high diets. No *Fusarium* species were isolated from the frass or the frass wheat crumb mixture. Larvae fed the four diets differing in the concentration of DON were analysed for multiple mycotoxins. The mycotoxins DON, 3-acetyldeoxynivalenol (3-ADON) and nivalenol (NIV) were the only mycotoxins detected in dried larvae and frass (Table 1). There was no difference in the level of DON detected in dried larvae between diets (*p* = 0.88). Similarly, 3-ADON in larvae did not differ among the diets although it should be noted that it was only detected in one replicate of larvae from each of the medium and high diets. The excretion of DON, 3-ADON and NIV was measured in a pooled sample of frass. Levels of DON in the frass were higher when compared to levels in the dried larvae within the low, medium and high diets (Table 1). Similarly, the 3-ADON levels detected in frass were higher than that detected in the dried larvae, including the control diet. Low levels of NIV were identified in frass from larvae fed the control and medium diets.

### 2.2. Feeding Trial and Yield Performance

In the feeding trial, survival was higher than 96%, the length of time until the first two pupae were observed in a replicate ranged from 32.2 ± 4.0 to 34.0 ± 2.7 days and the average daily gain (ADG) per worm ranged from 1.9 ± 0.1 to 2.1 ± 0.1 mg/day (Table 2). There was no effect of diet on any of these parameters (*p* > 0.05). A tendency was observed with protein efficiency ratio (PER; *p* = 0.05). The feed conversion ratio (FCR) differed between the control (2.1 ± 0.3) and low (2.9 ± 0.40) treatment (*p* < 0.01), and efficiency of conversion of ingested food (ECI) in mealworms fed the control (47.3 ± 6.0%) was higher than those on the low (35.4 ± 4.8%) or medium (38.0 ± 4.1) diets (*p* < 0.01).

### 2.3. Chemical Nutritional Analysis

The chemical nutritional composition of larvae reared on each of the four DON-contaminated diets plus the oven-dried control sample is reported in Table 3. The oven-dried larvae (ODL) and freeze-dried larvae (FDL) values were similar for the control diets. Larval crude protein (CP) values ranged from 49.4 to 51.9%, crude fat (CF) 34.4 to 36.0%, ash 3.2 to 3.6, acid detergent fiber (ADF) 5.2 to 5.7, acid detergent insoluble nitrogen (ADIN) 2.6 to 3.0 and chitin 2.7 to 2.9. Four fatty acids (FA) were detected (Table 4). Oleic acid (C18:1) was the most abundant with values between 55.0 to 56.9% of the total FA content. Linoleic acid (C18:2) had values ranging between 17.5 to 21.5%. Palmitic acid (C16:0) had slightly lower levels that ranged from 16.9 to 20.4%, whereas myristic acid (C14:0) was the least abundant FA detected, ranging from 5.2 to 7.0%. The ODL samples contained more myristic and palmitic acid than FDL but were lower in linoleic acid and oleic acid. The FAs palmitoleic acid (C16:l), stearic acid (C18:0), vaccenic acid (C18:l) γ-linoleic acid (C18:3), linolenic acid (C18:3), eicosenoic acid (C20: l), arachidonic acid (C20:4), eicosapentaenoic acid (C20:5), docosatetraenoic acid (C22:4), docosahexaenoic acid (C22:6) were analysed but were undetected in our samples. The amino acids detected in the larvae are reported in Table 5. Overall, amino acid values were the same among the treatments (including ODL).

### 2.4. Breeding and Preference Trials

In the first generation, survival of pupae from the control diet was higher (99.6 ± 0.6%) compared with larvae fed the low (98.2 ± 0.7%), medium (96.9 ± 0.9%) and high (98.4 ± 0.9%; *p* < 0.01) DON-contaminated diets (Table 6). The mortality observed did not exceed 3.5% for any of the diets. Beetle weights in the first-generation were 103.8 ± 8.1 mg for the control, and 101.5 ± 4.4 mg, 102.3 ± 5.4 mg, and 105.4 ± 4.3 mg for low, medium and high, respectively (Table 6). In the second-generation, beetle weight differed between diets (*p* < 0.01; Table 6). The average weight of 100 beetles was higher on the control (88.0 ± 4.3 mg) compared to the DON-contaminated diets low, medium and high (77.8 ± 1.5, 80.2 ± 3.3 and 76.0 ± 3.2 mg, respectively; Table 6). Adults from the second generation weighed less than those observed in the first. There were no differences between diets in terms of preference or avoidance (*p* = 0.07; data not shown).

## 3. Discussion

### 3.1. Mycotoxin Analysis

A major objective of this study was to determine the potential accumulation of mycotoxins in yellow mealworm larvae reared on *Fusarium*-damaged wheat with DON concentrations as high as 12,000 µg/kg. Our results demonstrated that after consuming high concentrations of DON for four weeks, DON was detected in the larvae, but did not exceed 131 µg/kg. Deoxynivalenol was not detected in larvae fed *F. culmorum*-infected wheat with 10,240 µg/kg of DON [10] nor in directly harvested or fasted larvae fed 4900 µg/kg (naturally contaminated) and 8000 µg/kg (spiked) DON [11]. In larvae the acetylated derivative 3-ADON was detected in concentrations up to 66 µg/kg. Camenzuli et al. [12] reported undetectable levels of 3-ADON in *Alphitobius diaperinus* and *Hermetia illucens* larvae fed diets spiked with DON, ZEN, aflatoxin B_1_ (AFB_1_) and ochratoxin A (OTA). Similarly, Van Broekhoven et al. [11] did not detect DON derivatives (15-ADON and DON-3G) in yellow mealworms fed diets containing these metabolites. In our study, DON and 3-ADON may have been detected due to partial metabolism of the high doses of DON and the conversion of it into 3-ADON via acetylation. Additionally, we exposed the larvae to higher levels of DON in naturally contaminated wheat and conducted a longer exposure (32–34 days) than the 15 days reported in the Guo et al. [10] and Van Broekhoven et al. [11] trials. Another reason for the detection of DON and 3-ADON may be due to a specific interaction between the larvae and the species of *Fusarium (F. graminearum)* contained in our diets as Guo et al. [10] noted that yellow mealworm larvae reacted differently to other species of *Fusarium.* Together, these factors could have facilitated the sequestration and therefore detection of DON and 3-ADON in the larvae.

In our samples, DON was partly excreted through faeces. The concentration of DON excreted was greater than that detected in the larval bodies, with those fed 12,000 ug/kg having the highest. The DON detected in the frass from larvae fed low and high diets was 15% and 6%, respectively, of the DON concentration in the wheat provided as diet. Van Broekhoven et al. [11] also fed a naturally-contaminated diet (4900 µg/kg DON) to yellow mealworm larvae and detected 14% of the consumed DON in frass. The DON derivative, 3-ADON, as well as NIV, were also detected in frass in our experiment and, as with DON, they were higher than the levels detected in the larvae or feed. Partial metabolism of DON to 3-ADON, or other metabolites (not analysed or unknown), may explain the appearance of these toxins in the frass. We believe that NIV was also present in larvae fed the low and high diets, but at concentrations below the limit of detection. While the mechanisms underlying DON metabolism and excretion in yellow mealworm larvae are still unclear, enzymatic activity by the mealworm’s midgut microbiota and the metabolic and/or enzymatic processes of the mealworm itself may contribute to the toxicokinetic of DON and other mycotoxins. Previous studies on the role of gut microbiota in the detoxification process in yellow mealworm larvae indicated a rapid adaptation of its microbial enzymatic package in response to dietary changes [13]. Therefore, it is possible that rearing conditions in the present study aided the adaptation of the gut microbiota in the larvae and facilitated the modification of DON into 3-ADON and other metabolites. Genta et al. [13] evaluated the activity of midgut enzymes and gut bacterial enzymes in the yellow mealworms and concluded that an interaction between the two aided the detoxification processes. Hence, microbial and midgut enzymes may lead to the acetylation of DON to 3-ADON and could explain why it was excreted at a higher concentration than that ingested. Since not all of the DON ingested from the diets was sequestered by larvae or excreted, it is possible that the remaining portion was metabolized into uncommon or unknown metabolites and were thus undetected in our samples. Processes other than acetylation convert DON into uncommon derivatives. For example, de-epoxidation (yielding DOM-1), glycosylation (yielding DON-3G, DON-15G, DON-3-O-glucuronide, DON-15-O-glucuronide), oxidation (yielding 3-oxo-DON), and epimerization (yielding 3-epi-DON) may be pathways used by our larvae to metabolize DON into novel derivatives [14]. Further studies into the enzymatic processes within mealworms fed DON-contaminated diets could provide insight into DON metabolism in insects and help guarantee the safety of mealworm protein for use in animal feed.

### 3.2. Mealworm Performance

The survival rate of yellow mealworm larvae reared on DON-contaminated wheat for 32–34 days was comparable to those reared on the control wheat, indicating that they could tolerate up to 12,000 ug/kg DON. Similar results were reported by Van Broekhoven et al. [11] where the survival rate was 98.3% for yellow mealworm larvae fed a natural DON-infected (4900 µg/kg) and 99.3% for those fed an artificially-contaminated diet (8000 µg/kg). Guo et al. [10] observed mortality greater than 20% after challenging the yellow mealworm larvae with different species of *Fusarium* (*F. graminearum* was not evaluated) with the highest mortality in larvae fed kernels infected with *F. bassiana* (59%) and *F. culmorum* (27%). The mortality in larvae exposed to *Fusarium*-contaminated wheat may depend not only on the mycotoxin(s) ingested, but also on the fungal species infecting the kernels [10,11].

Consuming concentrations of DON (up to 12,000 μg/kg) had no direct detrimental effects on yellow mealworm larval growth as weight and ADG were not different from larvae fed the control diet. Larvae fed the control wheat with 15% CP had a higher ECI value (47.3 ± 6.0%), and lower FCR (2.1 ± 0.3) compared to those fed the DON-contaminated diet with 11% CP. Van Broekhoven et al., [15] also found the highest ECI and lowest FCR values in yellow mealworm larvae were associated with high protein diets.

### 3.3. Chemical Nutritional Analysis

The dry matter and ash values in our samples were similar to those of other studies where larvae were fed wheat bran [9], wheat flour and yeast [16], or organic wastes [8,15]. In our study, larvae CP values were consistent across diets (50% protein content) and are similar to those previously reported for the yellow mealworms [9,15,16]. Larvae CF values in the current study were also similar to those previously reported by Ghaly and Alkoaik [16] and Ravzanaadii et al. [9]. Only four FA were detected in larvae, in the FDL oleic acid was the most prevalent followed by linoleic acid then palmitic acid and lastly myristic acid. The values of these four FA were similar to those reported by Oonincx et al. [17], Paul et al. [18] and Van Broekhoven et al. [15], except that we did not detect palmitoleic acid, stearic acid or α-linoleic acid. Low amounts of eicosenoic acid and vaccenic acid have been reported in yellow mealworm larvae by Ravzanaadii et al. [9]. Fasel et al. [19] studied the fatty acid composition of yellow mealworm larvae after feeding them different ratios and concentrations of ω-3/ω-6 FA. They concluded that the larvae possess plasticity to convert FA into triglycerides, and therefore changes in dietary lipids could be reflected in the FA composition of the larvae. Differences in the fatty acid profiles between our larvae and other studies may be a consequence of the different FA contained in our feed. Comparing the FAs detected in ODL and FDL, the lower linoleic acid level in the former could be the result of the higher temperature from the oven-drying method saturating it into palmitic and myristic acid (saturated fatty acids). The amino acid profiles in the dried larvae showed no major numerical differences. Our amino acid (dry matter) results were similar to those of other studies where yellow mealworm larvae were fed organic wastes [9,20].

Recent research has reported positive effects of chitin on animal health. For instance, chitin acted as an immunomodulator in the European sea bass (*Dicentrarchus labrax*) [21] and inhibited proliferation of microorganisms such as *Salmonella* and *Escherichia coli* in broilers [22]. For this reason, we wanted to determine whether or not chitin content would change after feeding larvae different levels of DON. Because chitin is structurally similar to cellulose, fiber in our samples was only estimated in the ADF fraction [23,24]. Our ADF, ADIN and chitin values were lower than those reported by Marono et al. [24], but higher than those found by Finke [25]. The variability between our study and these in terms of fiber suggested that mealworms may assimilate fiber according to the availability and quality in the protein (non-digestible nitrogen) source consumed. The data obtained after analysing these two drying methodologies suggests minimal differences in the nutrient profile of the larvae. However, of the two methods, the oven-drying method is likely more suitable based on scalability and cost when considering a large-scale mealworm production facility.

### 3.4. Breeding and Preference Trials

Evaluating survival, developmental time, and production traits in insects can be difficult as multiple factors such as small changes in the environment, availability and quality of feed, and intra-specific interactions can affect such parameters [26]. For example, low population densities have been associated with increased body weights in larvae, pupae and beetles and slow the instar change and pupation rates [26]. This may explain the difference we observed in pupation rate with an additional three days to the end point being observed in the smaller population (*n* = 4000) reared for the breeding trial compared to the larger population from the feeding trial (*n* = 6000; data not shown). There was no effect of diet on larvae and beetle survival or pupal and larval weight. We observed lower average pupal and beetle weight in the second-generation compared to the first generation. As previously discussed by Weaver and McFarlane [26] and observed here in our experiment, the increase in density from one generation to the next could have affected the biomass in these two life stages of the yellow mealworm. We aimed to determine whether or not yellow mealworm larvae preferred diets infected with *F. graminearum.* Unlike Guo et al. [10], we observed no difference in terms of preference or avoidance by yellow mealworm larvae to *F. graminearum*-infected and DON-contaminated diets.

In conclusion, yellow mealworm larvae reared on *Fusarium*-infected wheat diets containing up to 12,000 µg/kg DON accumulated a low level of DON (131 ± 23.6 μg/kg) and had minimal to no effect on larvae survival, nutritional composition and production traits. A broiler feeding trial is currently underway to investigate the effect of the inclusion of mealworm-meal from larvae reared on DON-contaminated wheat has on performance and health. Further research should investigate the effect of potentially sequestered DON-derivatives that may be produced by the larvae, and that could cause adverse effects in animals. These results support using DON-contaminated wheat in large-scale production of mealworms to produce a sustainable, safe protein source for animal feed.

## 4. Materials and Methods

### 4.1. Wheat Sources

Two sources of Canadian Western Red Spring wheat were purchased from grain producers in Saskatchewan, Canada. Mycotoxin concentration was determined using HPLC-tandem MS at Prairie Diagnostic Services (Saskatoon, Canada). One wheat source was found to initially contain 0 µg/kg and was subsequently used as the control wheat. The other wheat source contained DON at 8000 µg/kg. The 8000 µg/kg DON wheat was sorted using a BoMill IQ NIT grain sorter at Flaman Seed Cleaning and Handling Facility (Saskatoon, Canada) to concentrate the DON levels. This resulted in six quality-sorted fractions, from high to low quality with increasing levels of DON. The six fractions were re-analysed for DON levels (data not shown) and then combined to obtain three different concentrations of DON for the experimental diets: low, medium and high. The three diets and the control wheat were ground to ~1000-micron particle size using a hammer mill HMS.20X (Canyon City, USA) and retested for a panel of 14 mycotoxins. The final DON levels fed to mealworms were: 200 µg/kg (control), 2000 µg/kg (low), 10,000 µg/kg (medium) and 12,000 µg/kg (high). Additionally, analysis of dietary crude protein (CP) using Dumas-combustion method using a Leco FP-528 (St Joseph, MI, USA) revealed CP levels of 15.0%, 11.1%, 10.5% and 10.7% for control, low, medium, and high diets, respectively.

### 4.2. Fusarium Species Identification

Wheat kernels were surface-sterilized in 3% (*v*/*v*) NaOCl for 2 min and rinsed twice with water. One hundred kernels were randomly selected and plated on potato dextrose agar with 2 mg/L each of streptomycin, tetracycline and neomycin in petri dishes (10 seeds per dish). After 5–7 days at 25 °C under near UV light, *Fusarium* colonies were isolated and identified based on morphological characteristics [27]. Samples of frass (purified and a mixture with wheat crumbs/fines) were also cultured for *Fusarium* spp. to determine if the fungus could survive passage through the yellow mealworm larvae gastrointestinal tract. Samples were diluted to 1% and 2% (g/mL) with sterile water and 0.3 mL of each dilution was spread on potato dextrose agar surface. After seven days culturing at 25 °C under near UV light, fungal colonies were identified by examining morphological characteristics [27].

### 4.3. Yellow Mealworm Colony

Five hundred larvae and 50 beetles of *T. molitor* were purchased from Carolina Biological Supply (Burlington, MA, USA). Larvae and beetles were reared in large plastic containers (50 × 32 × 15 cm) with ground wheat (0 µg/kg DON) as the only source of food. Paper towels were laid on top of the wheat and spritzed with water three times per week. The room was maintained at 50% relative humidity, 25 °C and a 12-h light photoperiod. Pupae were moved into a separate plastic container twice weekly. Once beetles emerged they were placed into a new container.

### 4.4. Feeding Trial

A total of 6000 mealworms (7th to 9th instar) [28] were selected from the mealworm colony using a grain cleaning sieve 5.0 slotted mesh (1.98 × 19.05 mm). Three hundred mealworms were allocated per replicate and placed in plastic drawers (22.5 × 16.5 × 9 cm) containing 500 g of ground diet along with a paper towel laid on top that was spritzed with water three times per week. Each treatment was replicated five times.

Larvae were reared on one of the four diets until the first two pupae were observed in a replicate. The number of days to reach this endpoint, the pooled larval weight (minus any mortality) and the weight of the remaining feed was recorded to calculate feed intake (FI), ADG, FCR and ECI according to the formulas below. Survivability was assessed by daily counting of dead mealworms. After collecting the final weights, larvae were placed in an empty container to fast for 24 h and then washed with distilled water. The frass was collected and refrigerated at 4 °C and the larvae were euthanized by freezing at −20 °C. Protein efficiency ratio was calculated to determine protein quality of the diets and the efficiency of the larvae in converting protein from DON-contaminated wheat into body weight.

ADG = (Final weight – initial weight)/number of days on feed(1)

FCR = Weight of ingested feed/weight gained(2)

ECI = (Weight gained/weight of ingested feed) × 100%(3)

PER = Weight gained/total protein fed (dry basis)(4)

### 4.5. Breeding Trial

The breeding trial was a similar arrangement as the feeding trial except there were 200 mealworms per replicate, thus totalling 4000. Production data collected included the number of days until larvae reached the same endpoint as above, i.e., when two pupae were observed in a replicate, the development time required for 100 (50%) larvae to pupate and the average weight of the new pupae and new beetles. Survival was monitored in larvae, pupae and beetles by daily counting of dead. A second generation of larvae was generated to observe the effect of mycotoxins on body weight and survival in the offspring. Insects were moved to larger plastic containers and 500 g of additional diet was added. Upon accumulating 50 pupae, the weight of the pupae was recorded, along with the weight of 200 larvae and 100 beetles.

### 4.6. Preference Trial

To measure avoidance or attraction of mealworms to mycotoxin-contaminated diets, thirty naive larvae were randomly selected from the colony and allocated ten per replicate (*n* = 3). Each petri-dish (replicate) was divided into four quadrants containing 2 g of each of the four diets (control, low, medium and high). Each diet was placed at the outside edge of the petri dish within the quadrant. Thereafter, the ten mealworms were randomly selected and positioned in the centre of the petri-dish and left in a dark container without any disturbance. Thirty minutes later, the worms within each sector were counted. This process was repeated 20 times.

### 4.7. Mycotoxin Analysis

Mycotoxin concentration was tested in wheat, freeze dried larvae (FDL) and frass. Samples were analysed for 14 common mycotoxins at Prairie Diagnostic Services Inc., (Saskatoon, Canada) via ultra-high-performance liquid chromatography Agilent 1100, (Santa Clara, CA, USA) and mass spectrometry Micromass Quattro Ultima Platinum Mass Spectrometer, (Milford, MI, USA) [29]. The mycotoxin suite included DON and metabolites 3-ADON and 15-acetyldeoxynivalenol (15-ADON), NIV, ZEN, α-zearalenol, β-zearalenol, T-2 toxin, HT-2 toxin, diacetoxyscirpenol, OTA, fumonisin B_1_, fumonisin B_2_ and AFB_1_. Briefly, a 2.0 g ground test sample was combined with HPLC reagent grade acetonitrile (85%) plus 15% distilled-deionized water filtered through a Barnstead Nanopure water purification system. Following continuous stirring for 10 min, the mixture was filtered through Whatman 41 150 mm filter paper. The supernatant, a 3 mL volume, was filtered through a MycoSep 225 Trich cleanup cartridge. The sample was dried with nitrogen gas and reconstituted with 50% methanol/50% 10 mM aqueous ammonium acetate. The sample was filtered through a 0.45 um syringe. A 10 uL volume of the filtered material was added to 990 uL of the 85/15 acetonitrile/distilled-deionized filtered water. This final sample was injected into the LC/MS system. Mycotoxin testing in larvae was analysed per replicate while frass was assessed per treatment using a pooled sample from all replicates. Due to sample size limitations, duplicate samples were not prepared. The detection limits for all mycotoxins was 25 ug/kg with the exception of alpha and beta zearalenol, which had detection limits of 66 ug/kg.

### 4.8. Chemical Nutritional Analysis of Larvae

The moisture content of the larvae fed the four diets was determined after freeze-drying using a Labconco FreeZone 6 Liter Benchtop Freeze Dry System (Kansas City, MO, USA). An extra sample of larvae fed the control diet was oven-dried [30] (Method 930.15) to determine any differences in nutritional value between ODL and FDL. The ODL sample was dried at 60 °C overnight, then dry matter was analysed [30] (Method 930.15). To determine ash content, 2.2 g of dried sample (pooled from replicates) was ignited at 600 °C for 18 h [30] (Method 942.05). To analyse ADF, duplicates 0.5 g samples ground to a particle size of 1 mm were evaluated per treatment using an ANKOM^200^ fiber analyser [30] (Method 973.18). The remaining portion of the samples were used to estimate CP and obtain ADIN [24]. The chitin percentage was calculated from the ADF ash-free and ADIN values as described by Marono et al. [24]. Neutral detergent fiber was not assessed.

Crude protein was determined by two methods. For the Dumas-combustion method, 0.2 g of 1 mm ground dried larvae was placed in a gel capsule and combusted at 800 °C following the guideline of [30] (Method 990.03). Kjeldahl CP analysis [30] (Method 984.13) was performed as part of the ADIN analysis used for chitin estimation. The N conversion factor was 6.25 for both Dumas-combustion and Kjeldahl digestion methods. The amino-acid profile of larvae was determined by the nutrition lab of the Faculty of Agricultural and Food Sciences at University of Manitoba, (Winnipeg, Canada; [30] (Method 994.12) using amino acid analyser S2100 Sykam, (Eresing, Germany). To assess PER, a sample of each diet was analysed for CP by Dumas combustion and calculated as outlined in Gasco et al. [31].

Crude fat (CF) was assessed by the ethyl ether extraction gravimetric method [30] (method 920.39) using a Goldfish extraction apparatus model 3500 (Kansas City, USA). Larvae samples of 1.3 g were processed in duplicates per treatment and extracted for 16 h. For the fatty acid (FA) profile, lipids from the larvae samples (25 mg) were extracted and transesterified with 500 µL of 0.5 N alcoholic NaOH solution under nitrogen at 100 °C for 10 min. Residual fatty acids were then esterified by addition of 500 µL of BF_3_/methanol reagent under nitrogen at 100 °C for 30 min. After cooling to 30–40 °C, 2 mL of hexane was added and mixed. Saturated NaCl was then added and the layers were mixed and allowed to separate. The upper layer with fatty acid methyl esters (1 microL) was injected on an Agilent 6850 gas chromatograph complemented with an Agilent 7683 series autoinjector (Santa Monica, CA, USA). Fatty acids were detected by flame ionization after separation on a DB-17 column (30 m × 0.32 mm × 0.25 mm film thickness). Hydrogen was used as a carrier gas (4.3 mL/min; split ratio = 70:1) while nitrogen (40 mL/min) was used as the makeup gas. The detector temperature was 280 °C. The initial oven temperature (130 °C) was increased 5 °C/min to 175 °C then 60 °C/min to 280 °C after which it was held for 1.25 min. The total run time was 12 min per injection.

### 4.9. Statistical Analysis

Feeding and breeding trial data were analyzed as a randomized complete block design (RCBD). Replicates one and two from both the feeding and breeding trials were carried out concurrently in October 2016, while replicates three through five were conducted in November 2016. There were two blocks of two and three replicates when distributing the 6000 or 4000 mealworms between four diets with five replicates totaling 20 experimental units. The experimental unit was the replicate that had 300 or 200 mealworms. The statistical model used was: Y_ij_ = µ + P_i_ + α_j_ + e_ij_, where Y_ij_ was the dependent variable associated with block i and diet j; µ was the overall mean; P_i_ was the block effect associated with the two different time periods over which the trial was conducted; α_j_ was the fixed effect associated with the four diet treatments; and e_ij_ was the random error associated with observation ij.

The preference trial was assessed as a completely randomized design (CRD) following the model: Y_ij_ = µ + α_j_ + e_ij_, where model parameters were the same as described for the RCBD experiments. All experiments were analyzed using the MIXED procedure in SAS 9.4 (SAS Institute Inc., Cary, NC, USA). Data were normally distributed when analyzed by the Shapiro-Wilk test. Comparison of treatment means was assessed using the Tukey-Kramer HSD test at a *p* < 0.05 level of significance. Data obtained using pooled replicates were not statistically analyzed, but the values are reported.

## Figures and Tables

**Table 1:** The levels of mycotoxins detected in the diets, dried mealworms, and frass (µg/kg) of yellow mealworm larvae fed four different levels of deoxynivalenol (DON)-contaminated wheat.

Mycotoxin	Treatments	Diets	Mealworm ^1^	Frass ^2^
DON	Control	200	136 ± 40.5	131
	Low	2000	127 ± 30.5	324
	Medium	10,000	122 ± 19.3	230
	High	12,000	131 ± 23.6	742
3-ADON	Control	<LOD	66 *	286
	Low	63	66 *	323
	Medium	52	66 *	326
	High	205	65 *	280
NIV	Control	<LOD	<LOD	50
	Low	<LOD	<LOD	<LOD
	Medium	<LOD	<LOD	51
	High	<LOD	<LOD	<LOD

^1^ Mean of replicates (*n* = 5) per treatment ± standard deviation; ^2^ pooled samples from five replicates; * detectable only in 1 to 3 replicates; <LOD: below limit of detection; limit of detection for DON; 3-ADON and NIV was 25.0 µg/kg; DON deoxynivalenol; 3-ADON 3-acetyldeoxynivalenol and NIV nivalenol.

**Table 2:** Production parameters, performance and survival of yellow mealworm larvae fed four different levels of deoxynivalenol (DON)-contaminated wheat.

Parameter	Treatments ^1^				SEM ^2^	*p* Value
	Control	Low	Medium	High		
Days to endpoint	32.2 ± 4.0	33.6 ± 3.3	34.0 ± 2.7	34.0 ± 2.7	1.45	0.79
Survival (%) ^3^	96.4 ± 2.5	97.7 ± 0.8	96.7 ± 1.7	98.0 ± 1.9	2.43	0.47
Larval weight (mg) ^4^	93.3 ± 8.4	99.9 ± 9.8	101.9 ± 7.4	100.2 ± 10.4	4.05	0.47
ADG (mg/day) ^4^	1.9 ± 0.1	2.1 ± 0.1	2.1 ± 0.1	2.0 ± 0.1	0.04	0.17
FCR ^4^	2.1 ± 0.3 _a_	2.9 ± 0.4 _b_	2.7 ± 0.3 _ab_	2.5 ± 0.1 _ab_	0.13	<0.01
ECI (%) ^4^	47.3 ± 6.0 _a_	35.4 ± 4.8 _b_	38.0 ± 4.1 _b_	40.0 ± 2.0 _ab_	2.01	<0.01
PER ^3^	3.1 ± 0.4	3.2 ± 0.4	3.6 ± 0.4	3.7 ± 0.2	0.17	0.05

^1^ Mean of replicates (*n* = 5) per treatment ± standard deviation; ^2^ SEM (standard error of the means); ^3^ trait evaluated per 300 larvae; ^4^ traits evaluated per individual larvae; ADG (average daily gain); FCR (feed conversion ratio); ECI (efficiency of conversion of ingested food); PER (protein efficiency ratio); values within a row that are followed by a different letter indicates significant differences (*p* < 0.05) and concentration of DON in diets: 200 µg/kg (control), 2000 µg/kg (low), 10,000 µg/kg (medium) and 12,000 µg/kg (high).

**Table 3:** Chemical nutritional analysis of yellow mealworm larvae fed different four different levels of deoxynivalenol (DON)-contaminated wheat (dry matter percent).

Parameter	Treatments				
	ODL	FDL			
	Control	Control	Low	Medium	High
Dry Matter	39.7	39.9	41.0	42.4	40.1
Crude Protein	50.2	51.9	49.4	50.7	49.6
Crude Fat	34.4	34.7	36.0	35.5	35.2
Ash	3.2	3.6	3.4	3.4	3.5
ADF	5.7	5.3	5.2	5.6	5.4
ADIN	3.0	2.6	2.6	2.7	2.6
Chitin	2.8	2.7	2.7	2.9	2.7

ODL (oven-dried larvae); FDL (freeze-dried larvae); ADF (acid detergent fiber); ADIN (acid detergent fiber insoluble nitrogen); concentration of DON in treatments: 200 µg/kg (ODL fed control), 200 µg/kg (FDL fed control), 2000 µg/kg (low), 10,000 µg/kg (medium) and 12,000 µg/kg (high).

**Table 4:** Fatty acid profile of yellow mealworm larvae fed four different levels of deoxynivalenol (DON)-contaminated wheat (dry matter percent).

Fatty Acid			Treatments				
			ODL	FDL			
Common Name	Lipid Number	ω-n	Control	Control	Low	Medium	High
Myristic acid	C14:0		7.0	5.6	5.2	5.3	5.3
Palmitic acid	C16:0		20.4	16.9	18.1	18.2	18.6
Oleic acid	C18: l	ω-9	55.1	56.9	55.5	55.0	55.3
Linoleic acid	C18:2	ω-6	17.5	20.6	21.3	21.5	20.8

ODL (oven-dried larvae); FDL (freeze-dried larvae); concentration of DON in treatments: 200 µg/kg (ODL fed control), 200 µg/kg (FDL fed control), 2000 µg/kg (low), 10,000 µg/kg (medium) and 12,000 µg/kg (high).

**Table 5:** Amino acid content of yellow mealworm larvae fed four different levels of deoxynivalenol (DON)-contaminated wheat (dry matter percent).

Amino Acid	Treatments				
	ODL	FDL			
	Control	Control	Low	Medium	High
Aspartic Acid	4.2	4.3	4.1	4.2	4.3
Threonine	2.1	2.1	2.1	2.1	2.1
Serine	2.6	2.7	2.6	2.6	2.7
Glutamic Acid	6.2	6.0	5.8	5.9	6.0
Proline	3.3	3.4	3.3	3.4	3.4
Glycine	2.7	2.8	2.8	2.8	2.8
Alanine	4.0	4.1	4.1	4.1	4.2
Cysteine	0.4	0.5	0.5	0.5	0.5
Valine	3.0	2.9	3.0	3.0	3.0
Methionine	0.6	0.7	0.6	0.7	0.7
Isoleucine	2.0	2.0	2.1	2.1	2.1
Leucine	3.7	3.7	3.7	3.7	3.7
Tyrosine	4.0	3.8	3.8	3.9	3.9
Phenylalanine	1.9	1.9	1.8	1.9	1.9
Histidine	4.8	5.0	4.8	4.8	5.1
Lysine	2.6	2.7	2.7	2.8	2.7
Arginine	2.6	2.7	2.7	2.7	2.7
NH_3_	0.7	0.7	0.7	0.7	0.7

ODL (oven-dried larvae); FDL (freeze-dried larvae); concentration of DON in treatments: 200 µg/kg (ODL fed control), 200 µg/kg (FDL fed control), 2000 µg/kg (low), 10,000 µg/kg (medium) and 12,000 µg/kg (high).

**Table 6:** Development traits of yellow mealworm larvae, pupae, and beetles fed four different levels of deoxynivalenol (DON)-contaminated wheat.

Parameter	Treatments ^1^				SEM ^2^	*p* Value
	Control	Low	Medium	High		
**First generation**						
Time to endpoint (days)	36.6 ± 1.3	35.2 ± 0.5	35.0 ± 1.9	35.0 ± 2.4	0.74	0.38
Time to pupation (days)	50.6 ± 2.1	52.4 ± 1.8	53.0 ± 1.9	50.4 ± 2.3	0.91	0.15
Larvae survival (%)	98.4 ± 0.7	99.3 ± 1.1	99.0 ± 1.2	99.1 ± 1.3	0.49	0.61
Pupal survival (%)	99.6 ± 0.6 _a_	98.2 ± 0.7 _b_	96.9 ± 0.9 _b_	98.4 ± 0.9 _b_	0.36	<0.01
Adult survival (%)	96.6 ± 3.8	96.6 ± 4.1	97.0 ± 2.1	97.8 ± 1.2	1.35	0.91
Pupal weight (%)	125.5 ± 2.5	123.3 ± 5.0	123.7 ± 4.7	127.1 ± 3.3	1.93	0.41
Adult weight (mg)	103.8 ± 8.1	101.5 ± 4.4	102.3 ± 5.4	105.4 ± 4.3	2.57	0.72
**Second generation**						
Larval weight (mg)	121.3 ± 4.2	119.7 ± 4.0	116.1 ± 3.8	119.5 ± 5.4	1.97	0.33
Pupal weight (mg)	109.6 ± 3.3	106.0 ± 6.9	105.2 ± 3.0	106.0 ± 7.8	2.53	0.62
Beetle weight (mg)	88.0 ± 4.3 _a_	77.8 ± 1.5 _b_	80.2 ± 3.3 _b_	76.0 ± 3.2 _b_	1.45	<0.01

^1^ Mean of replicates (*n* = 5) per treatment ± standard deviation; ^2^ SEM (standard error of the means); values within a row that are followed by a different letter indicate significant differences (*p* < 0.05); concentration of DON in diets: 200 µg/kg (control), 2000 µg/kg (low), 10,000 µg/kg (medium) and 12,000 µg/kg (high).
